# MISEV2023: An updated guide to EV research and applications

**DOI:** 10.1002/jev2.12416

**Published:** 2024-02-23

**Authors:** Joshua A. Welsh, Deborah C. Goberdhan, Lorraine O'Driscoll, Clotilde Théry, Kenneth W. Witwer

**Affiliations:** ^1^ Translational Nanobiology Section, Laboratory of Pathology, National Cancer Institute National Institutes of Health Bethesda Maryland USA; ^2^ Nuffield Department of Women's and Reproductive Health University of Oxford, Women's Centre, John Radcliffe Hospital Oxford UK; ^3^ School of Pharmacy and Pharmaceutical Sciences Trinity College Dublin Dublin Ireland; ^4^ Trinity Biomedical Sciences Institute Trinity College Dublin Dublin Ireland; ^5^ Trinity St. James's Cancer Institute Trinity College Dublin Dublin Ireland; ^6^ Institut Curie, INSERM U932 PSL University Paris France; ^7^ CurieCoreTech Extracellular Vesicles Institut Curie Paris France; ^8^ Department of Molecular and Comparative Pathobiology Johns Hopkins University School of Medicine Baltimore Maryland USA; ^9^ EV Core Facility “EXCEL”, Institute for Basic Biomedical Sciences Johns Hopkins University School of Medicine Baltimore Maryland USA; ^10^ The Richman Family Precision Medicine Center of Excellence in Alzheimer's Disease Johns Hopkins University School of Medicine Baltimore Maryland USA

## 1 MISEV2023: DELIVERING THE GOODS

MISEV2023 has been written and revised to stand on its own as a guide to EV studies . Starting with an introduction with a historical perspective and ‘user guide’, MISEV2023 next takes the reader through all of the main aspects of an EV study. Community‐sourced, it has something to offer beginners and veterans alike. The **nomenclature** section introduces the diversity of EVs and the need to avoid misusing the term ‘exosome’. It also highlights the ‘non‐vesicular extracellular particles’ (NVEPs) that abound in most EV preparations. **Collection and pre‐processing** underscores the importance of pre‐analytical variables, with input on specific EV sources from ISEV task forces. **EV separation and concentration** and **EV characterization** have new details on key approaches. A new section on **technique‐specific reporting for EV characterization** focuses on leading commercially available methods. **EV release and uptake** examines opportunities and pitfalls, while **functional studies** reminds us of important controls that are needed to help attribute an outcome to EVS. Finally, there is a new section on **studying EVs in vivo**.

## 2 MISEV2023: CONTINUING A STRONG TRADITION

Despite the stand‐alone nature of MISEV2023, it is nevertheless instructive to read and interpret this new document in the context of the previous MISEV publications, MISEV2014 and MISEV2018 (Lötvall et al., 2014; Théry et al., 2018). MISEV2014 was an editorial of the ISEV board, while MISEV2018 first gathered community input, with nearly 400 contributors. Interspersed with the previous MISEVs, numerous ISEV position papers, along with collaborative reviews and editorials, have guided the field in important ways. The ISEV position papers are summarized in Table 1 of MISEV2023.

**TABLE 1:** MISEV authors and countries.

Country	Number of authors	MISEV authors per million^a^
USA	276	0.812
Australia	71	2.685
Italy	67	1.138
UK	59	0.871
France	56	0.865
Germany	50	0.600
Canada	43	1.109
Netherlands	37	2.100
Sweden	32	3.015
Spain	32	0.673
Japan	29	0.235
Hungary	28	2.915
Korea	22	0.425
China	22	0.015
Belgium	20	1.711
Austria	17	1.898
Switzerland	16	1.819
India	15	0.010
Denmark	14	2.369
Brazil	14	0.065
Portugal	13	1.269
Ireland	12	2.373
Slovenia	11	5.189
Poland	11	0.268
New Zealand	9	1.721
Finland	9	1.623
Norway	8	1.461
Chile	6	0.306
Czech Republic	5	0.476
Argentina	5	0.109
Taiwan	4	0.167
Malaysia	4	0.117
Singapore	3	0.499
Israel	3	0.327
Kazakhstan	3	0.153
Iran	3	0.034
Luxembourg	2	3.055
Estonia	2	1.512
Serbia	2	0.280
South Africa	2	0.033
Turkey	2	0.023
Iceland	1	2.664
Malta	1	1.869
Cyprus	1	0.794
Latvia	1	0.546
Lithuania	1	0.368
Uruguay	1	0.292
Costa Rica	1	0.192
Thailand	1	0.014
Egypt	1	0.009
Mexico	1	0.008
Russia	1	0.007
Bangladesh	1	0.006

^a^ Country populations as indicated by Wikipedia (https://en.wikipedia.org/wiki/List_of_countries_and_dependencies_by_population, accessed December 13, 2023 and based mostly on United Nations sources).

## 3 MISEV2023: THE PROCESS

MISEV2023 began, as did MISEV2018, with a pre‐drafting survey (Witwer et al., 2021). The input from this 2020 survey identified existing aspects to emphasize and new areas to explore. After reviewing the results of the survey, the ISEV board met at the end of 2020 and assigned a five‐author committee (J.A. Welsh, D.C. Goberdhan, L. O'Driscoll, C. Théry and K.W. Witwer) to prepare a draft and guide the community input for the next MISEV. Numerous drafts and refinements were then prepared and shared with the ISEV board for feedback. Approximately 70 authors contributed to section drafts during this phase. Then, in 2022, an intermediate draft and a survey were shared with the ISEV community, resulting in >1000 responses. The three co‐first authors worked hard and long to update MISEV based on this input. Multiple rounds of internal review and additional invited contributions/revisions ensued, along with style ‘unification’. In mid‐2023, the article received pre‐submission comments from JEV, followed by more revisions, formal submission, and three post‐submission rounds of scrutiny (the first, main round including comments from more than 30 individuals). A near‐final draft was shared with all co‐authors for confirmation and a consensus survey. MISEV2023 was formally approved for publication by JEV on 13 December 2023.

## 4 WITH GRATITUDE TO ALL CONTRIBUTORS

**FIGURE 1:**
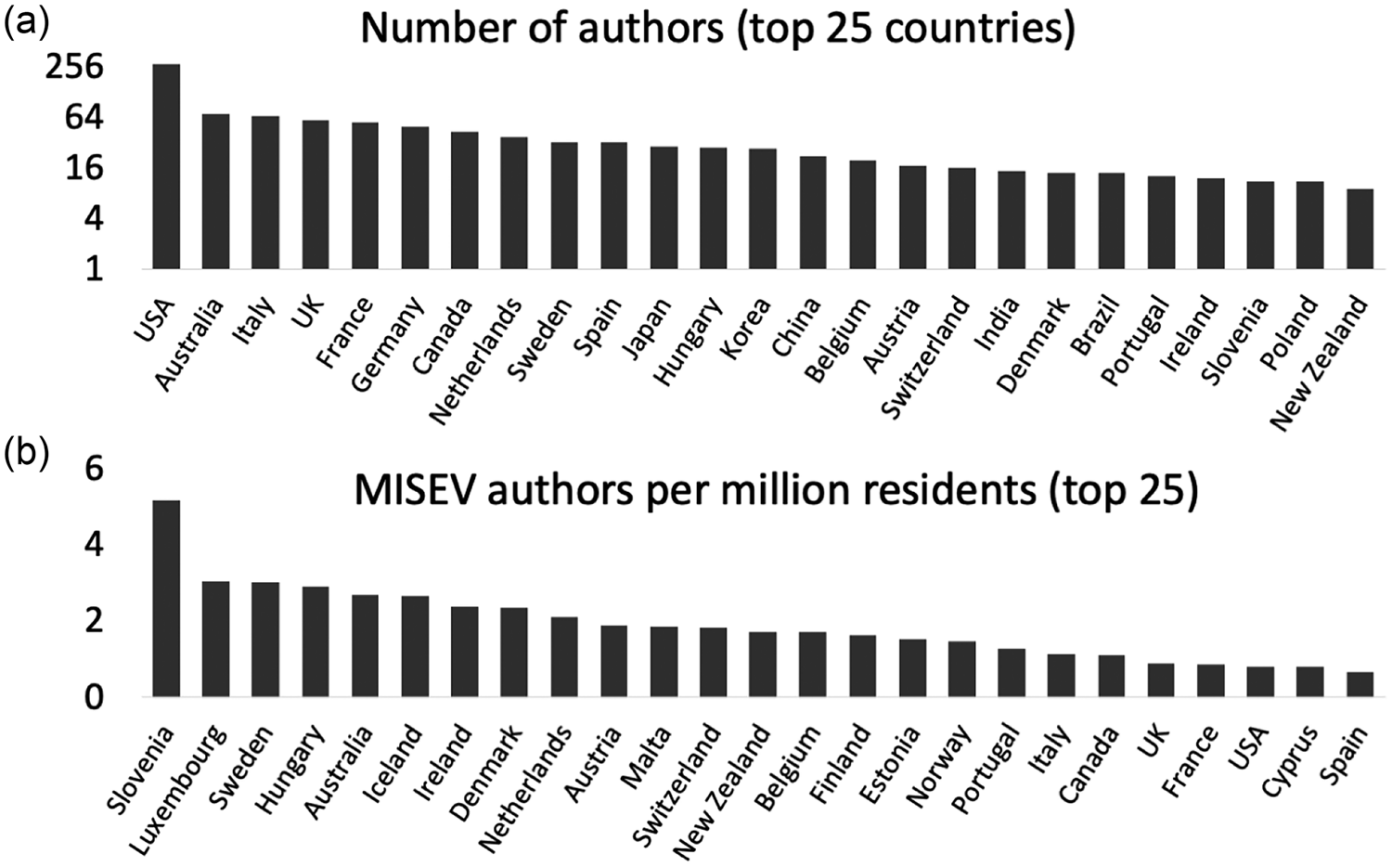
MISEV authors by country. (a) The total number of MISEV authors per country for the top 25 countries. Counts are based on country of primary (first listed) affiliation as provided by each individual author. (b) Number of MISEV authors per capita (millions) for the top 25 most ‘dense’ contributing countries. Country populations as indicated by Wikipedia (https://en.wikipedia.org/wiki/List_of_countries_and_dependencies_by_population, accessed December 13, 2023 and based mostly on United Nations sources).

MISEV2023 has been a community effort like none other in the history of ISEV. Seventy four scientists contributed original text and are listed on the ‘front page’ of the article as drafting authors. The final manuscript is published with 1051 co‐authors, almost all of whom completed several extensive feedback questionnaires. According to primary affiliations, the authors represent at least 53 countries (Figure 1a, Table 1), with the top five including at least one country from each of the three geographical chapters of ISEV: the United States, Australia, Italy, the United Kingdom and France. The highest density of authors (per capita) was achieved by Slovenia, followed by Luxembourg, Sweden, Hungary and Australia (Figure 1b). The average author spent multiple hours completing the 2022 MISEV survey. Likewise, the average author spent more than 2 h completing the 2023 authorship confirmation and consensus survey. We cannot possibly know the total amount of time that authors invested in MISEV2023, but we do know that, for most authors, the in‐survey hours were accompanied by even more time spent reading and considering the manuscript before survey completion. Our debt of gratitude to the authors is tremendous.

## 5 LIMITATIONS

Among the many strengths of MISEV2023, there are also limitations. We acknowledge that MISEV is not and has never aimed to be a be‐all and end‐all document for EV research and translation. This is addressed in a dedicated introductory section ‘what MISEV “IS” and “IS NOT”’. Here, we clarify that MISEV should help build rigor and reproducibility and serve as a source of knowledge to help educate newcomers to the EV field. MISEV should not be an unreasonable barrier to entry, nor should it stifle innovation. While a number of sections focus on mammalian EVs, MISEV principles are generally relevant for EVs from all sources. We also highlight that MISEV is highly pertinent to translational and therapeutic applications: its approaches help to establish that a given biomarker or therapeutic outcome is actually associated with or facilitated by EVs.

## 6 MISEV AND THE EV LITERATURE

Similarly, MISEV is by no means a comprehensive review of the literature. As indicated in the introduction, not every one of the >500 citations in the paper is necessarily the first or the best paper to support the related statement. Most citations were suggested by the drafting topic experts, while others were added based on the community survey and reviewer comments. Compared with the first draft that was shared with the community in 2022, the published version of MISEV has around 200 added citations. Despite attempts to unify approaches, some MISEV sections have different citation density and perhaps even citation quality from others. Indeed, not all cited papers are necessarily consistent with the current guidelines. A citation is not endorsement of the approaches or conclusions of any study, and lack of citation also does not necessarily mean that a specific paper has been evaluated and rejected. We thank the community for their citation suggestions and apologize for any omissions and not doing full justice to the literature. At the time of the MISEV2014 editorial, it was still possible for an individual EV expert to maintain a reasonably comprehensive overview of the EV literature, which was limited to a few hundred key primary research publications. By MISEV2018, this situation had ended. Currently, thousands of EV publications appear each year, hence the challenge of categorizing their primacy and quality.

## 7 BRING IT ON: CRITIQUE AND DEBATE ARE GOOD FOR EV SCIENCE

MISEV is widely accepted and useful, but it is not perfect. Admittedly, a large portion of MISEV could be replaced by one statement: ‘Tell us exactly what you did’. We hope and trust, though, that the text going beyond this is nevertheless helpful for readers and not overly dogmatic. Every large scientific enterprise will have its detractors. But ultimately, most community members have been highly supportive of the MISEV process and product. We invite the remaining skeptics to follow the example of some of our harshest and most principled critics, who vociferously shared their viewpoints and objections, thereby helping make MISEV into a better document. Together, we can do better!

## 8 WHAT NEXT: MISEV2028?

Does doing better mean a future MISEV? MISEV2018 followed MISEV2014 by 4years, and MISEV2023 has now been released another 5years later. Will there be a need for another MISEV? The process of delivering MISEV has become complicated and lengthy, perhaps prohibitively so. MISEV2014 was essentially an editorial, written and published by a handful of scientists in a matter of months. MISEV2018 was more involved, introducing community engagement and taking a year from initial decisions to publication. MISEV2023 has been a much longer journey of 3years. The full manuscript went through more than 30 distinct drafts. Despite the reward of the final outcome, would we have agreed to this assignment if we had known the true time commitment? In future, it may be difficult to expect any volunteer scientist to devote intensive, unpaid effort for 3or more years to a project of this magnitude.

Before any future iteration of MISEV, we would encourage careful evaluation of whether an update is truly needed, and, if so, a more structured approach to its construction. It may be more helpful for future guidelines to focus on specific methods, reagents, experimental design, or applications without a field‐wide renewal of MISEV. These products could be led by ISEV task forces and special interest groups or ad hoc committees or collaborations with partner organizations. Perhaps MISEV could also be moved to a ‘living document’, such as a wiki‐style approach, in which different parts can be updated as often and as intensively as thought necessary: a MISEV of the minute, not the half‐decade. New tools, including artificial intelligence, if they continue to develop and are available for future MISEV‐related initiatives, might be used to craft stronger consensus and, for example, better identify the ‘right’ citations than any human crowd‐sourced document. Whatever is decided from the outset, our experience suggests that careful definition of the overall process and clear delegation of decision‐making will be important.

## 9 FOR NOW: WORKING TOGETHER FOR MISEV AWARENESS AND DISSEMINATION

Starting with the pre‐MISEV survey in 2020 and continuing today post‐acceptance, more than 100 community members have volunteered to help spread the word about MISEV2023. Some scientists intend to write editorials or perspectives articles for journals they are familiar with. Others will reach out to journal editors and publishers to ensure that they are aware of MISEV2023. Several co‐authors plan to feature MISEV2023 at a journal club or in a session at a scientific meeting, while others hope to contact regulatory entities or scientific societies. A press release has also been prepared. We are grateful for any and all assistance in bringing the value of MISEV to the widest audience possible and thereby elevating the entire EV community.

***In conclusion*,** we hope that the ultimate goal of MISEV will continue to be achieved: guiding the EV community to forge better understanding of basic biology and translate findings into benefits for human health and wider applications. Let us please take the opportunity to renew our commitment to this goal and to each other. Thank you for the opportunity to serve. It has been a pleasure to work with the vibrant EV community and coordinate our combined efforts into MISEV2023.

## CONFLICT OF INTEREST STATEMENT

The authors declare no conflicts of interest.

## References

[jev212416-bib-0001] Lötvall, J. , Hill, A. F. , Hochberg, F. , Buzás, E. I. , Di Vizio, D. , Gardiner, C. , Gho, Y. S. , Kurochkin, I. V. , Mathivanan, S. , Quesenberry, P. , Sahoo, S. , Tahara, H. , Wauben, M. H. , Witwer, K. W. , & Théry, C. (2014). Minimal experimental requirements for definition of extracellular vesicles and their functions: A position statement from the International Society for Extracellular Vesicles. Journal of Extracellular Vesicles, 3, 26913. 10.3402/jev.v3.26913 25536934 PMC4275645

[jev212416-bib-0002] Théry, C. , Witwer, K. W. , Aikawa, E. , Alcaraz, M. J. , Anderson, J. D. , Andriantsitohaina, R. , Antoniou, A. , Arab, T. , Archer, F. , Atkin‐Smith, G. K. , Ayre, D. C. , Bach, J.‐M. , Bachurski, D. , Baharvand, H. , Balaj, L. , Baldacchino, S. , Bauer, N. N. , Baxter, A. A. , Bebawy, M. , … Zuba‐Surma, E. K. (2018). Minimal information for studies of extracellular vesicles 2018 (MISEV2018): A position statement of the International Society for Extracellular Vesicles and update of the MISEV2014 guidelines. Journal of Extracellular Vesicles, 7, 1535750. 10.1080/20013078.2018.1535750 30637094 PMC6322352

[jev212416-bib-0003] Welsh, J. A. , Goberdhan, D. C. I. , O’Driscoll, L. , Buzas, E. I. , Blenkiron, C. , Bussolati, B. , Cai, H. , Di Vizio, D. , Driedonks, T. A. P. , Erdbrügger, U. , Falcon‐Perez, J. M. , Fu, Q. , Hill, A. F. , Lenassi, M. , Lim, S. K. , Mahoney, M. G. , Mohanty, S. , Möller, A. , … Nieuwland, R. (2024). Minimal information for studies of extracellular vesicles (MISEV2023): From basic to advanced approaches. Journal of Extracellular Vesicles, 13(2). e12404. 10.1002/jev2.12404 38326288 PMC10850029

[jev212416-bib-0004] Witwer, K. W. , Goberdhan, D. C. , O'Driscoll, L. , Théry, C. , Welsh, J. A. , Blenkiron, C. , Buzás, E. I. , Di Vizio, D. , Erdbrügger, U. , Falcón‐Pérez, J. M. , Fu, Q. , Hill, A. F. , Lenassi, M. , Lötvall, J. , Nieuwland, R. , Ochiya, T. , Rome, S. , Sahoo, S. , & Zheng, L. (2021). Updating MISEV: Evolving the minimal requirements for studies of extracellular vesicles. Journal of Extracellular Vesicles, 10, 00. 10.1002/JEV2.12182 PMC8710080 34953156

